# Changes in the Murine Microbiome and Bacterial Extracellular Vesicle Production in Response to Antibiotic Treatment and Norovirus Infection

**DOI:** 10.3390/v15122443

**Published:** 2023-12-16

**Authors:** Chanel A. Mosby, Kendall J. Long, Matthew B. Phillips, Julia Bartel, Melissa K. Jones

**Affiliations:** 1 Microbiology and Cell Science Department, IFAS, University of Florida, Gainesville, FL 32611, USA; c.mosby.haundrup@ufl.edu (C.A.M.); kendall.long@ufl.edu (K.J.L.); jbartel@ufl.edu (J.B.); 2 Molecular Genetics and Microbiology Department, College of Medicine, University of Florida, Gainesville, FL 32611, USA; mphillips1@ufl.edu

**Keywords:** murine norovirus, bacterial extracellular vesicles, microbiome composition, 16S sequencing, *Lachnospiraeae*, outer membrane vesicle, OMV, bEV, MNV infection

## Abstract

Norovirus infection is influenced by the presence of commensal bacteria, and both human and murine norovirus (MNV) bind to these bacteria. These virus–bacterial interactions, as well as MNV infection, promote the increased production of bacterial extracellular vesicles (bEVs). However, no correlation has been made between specific bacterial groups, their vesicles, and their impact on norovirus infection. The current study evaluated the impact of select bacterial compositions of murine microbiomes using antibiotic (ABX) cocktails on MNV infection and bEV production. The goal of this research was to determine if increases in bEVs following MNV infection in mice were associated with changes in specific bacterial populations. Bacterial taxa were found to be differentially abundant in both ABX-treated and untreated mice, with the greatest change in bacterial taxa seen in mice treated with a broad-spectrum ABX cocktail. Specifically, *Lachnospiraeae* were found to be differentially abundant between a variety of treatment factors, including MNV infection. Overall, these results demonstrate that MNV infection can alter the abundance of bacterial taxa within the microbiota, as well as their production of extracellular vesicles, and that the use of selective antibiotic treatments can allow the detection of viral impacts on the microbiome that might otherwise be masked.

## 1. Introduction

Human norovirus (HNoV) is the leading cause of acute gastroenteritis in the United States for people of all ages [1,2]. Infection by HNoV occurs most commonly when infected feces, food, or fluid are ingested [3], revealing its highly contagious nature. HNoV has been previously found to bind to commensal bacteria [1], raising questions as to how this may impact viral infection. Murine norovirus (MNV) is an alternate strain of norovirus that similarly binds to commensal bacteria [4], and the presence of commensal bacteria has been shown to alter MNV replication along the intestinal tract in a region-specific manner [5]. In fact, the MNV model system has been utilized to better understand the impacts of norovirus interactions with commensal bacteria and how these interactions influence viral infection. In addition to commensal bacteria influencing norovirus infection, the ability of the virus to alter the composition of the intestinal microbiome has also been examined. While one study using MNV has shown that some differences in microbiome composition can occur between mock and MNV-infected mice at later timepoints (5 days post-infection) [6], others have found few changes in composition at the peak of viral replication [7].

While it is established that commensal bacteria influence norovirus infection, the precise mechanisms through which this occurs are currently unknown [5,8,9]. Research examining the role of commensal bacteria in other enteric viral infections has found that the lipopolysaccharide (LPS) of Gram-negative bacteria, as well as additional bacterial protrusions, increases viral replication by enhancing both viral stability, replication, and environmental fitness [10,11]. For MNV, bile acids are involved in the suppression of viral replication in the proximal regions of the small intestine, suggesting a link to specific bacterial species that metabolize host bile acids, yet these bacteria have yet to be identified [8].

In addition to bile acids, another bacterial byproduct shown to influence MNV replication is outer membrane vesicles (OMVs), where co-infection with these vesicles leads to the suppression of MNV replication in macrophages in vitro [12]. Interestingly, similar to what is observed during in vitro interactions between noroviruses and commensal bacteria in vitro, MNV infection of mice leads to increased vesicle production by the intestinal microbiome, indicating that these vesicles may play a role in regulating viral infection [13]. However, as with bile acids, the specific bacterial species associated with MNV-induced vesicle production have not been identified.

The study described herein used antibiotics to selectively deplete targeted bacterial populations in the intestinal tract to determine the influence of the remaining bacterial population on MNV infection and MNV-induced bacterial extracellular vesicle (bEV) production. The microbiome was also analyzed both pre- and post-MNV infection to further assess the impact of MNV infection on specific bacterial populations. The results showed that MNV infection resulted in the differential abundance of bacterial taxa and altered bEV production.

## 2. Materials and Methods

### 2.1. Murine Norovirus Production

Recombinant murine norovirus-1 (MNV-1) was generated with plasmid pSPMNV-1.CW3 (provided by Dr. Stephanie Karst) as previously described [14]. In summary, 293T cells were transfected with 5 micrograms of pSPMNV-1.CW3. The cells were harvested after 24 h and then lysed. The supernatant was then collected and centrifuged. RAW264.7 cells were infected with the clarified supernatant (0.05 MOI) and harvested at 36–48 hpi. Cells were lysed by freeze–thaw and the clarified supernatant underwent ultracentrifugation through a 25% sucrose cushion before the virus pellet was resuspended in dPBS. The viral titer was quantified using the TCID_50_ assay. All viral stocks were stored at −80 °C.

### 2.2. Mice Infections and Antibiotic Treatment

The C57BL/6 mice used in this study were bred and housed in the University of Florida animal facilities. The animal protocols were approved by the Institutional Animal Care and Use Committees at the University of Florida. In all experiments, age- and sex-matched groups were used. Mice were 9 to 15 weeks of age, and comparable numbers of male and female mice were used in each experiment. Sample sizes were determined based on our previous experiences with MNV mice infection studies.

The intestinal microbiota was depleted from mice as described previously [5]. Briefly, mice were administered a daily cocktail of the indicated antibiotic(s) or PBS control (Table 1). After five days, the antibiotics were added to the drinking water. To confirm efficient depletions, stool samples were collected from the mice after the fifth day. The stool samples were homogenized and plated on brain–heart infusion agar with 10% sheep blood before incubation under anaerobic and aerobic conditions at 37 °C for 2 days. Antibiotics or PBS were maintained in the drinking water for these two days and the infections were carried out only after the antibiotic-treated animals were confirmed to be free of the indicated bacteria by plating on nutritive media under aerobic and anaerobic conditions. On the seventh day, mice were orally inoculated with 10^7^ MNV-1 or mock inoculum. Stool samples were collected just prior to MNV infection (pre-challenge) and 24 h post-infection before being stored at −80 °C. After stool sample collection, mice were euthanized, and the viral titers of the intestines were analyzed.

### 2.3. Stool DNA Extraction and Sequencing

Forty-five mg of stool was used for DNA extraction using the E.Z.N.A Stool Extraction Kit (Omega Bio-tek, Norcross, GA, USA) and following manufacturer instructions. To reduce batch bias during extraction, samples were randomized. Negative controls were treated as samples during extraction to control for the potential contamination of kit components with additional negative controls added in the PCR steps. Extracted DNA was then quantified using a 1X dsDNA High Sensitivity Kit with a Qubit 2.0 fluorometer (Invitrogen, Waltham, MA, USA) and assayed for purity using a Nanodrop spectrophotometer (Thermo Scientific, Waltham, MA, USA). Fifty ng of extracted DNA was then used for PCR to amplify the V3–V4 variable region of the 16S rRNA gene using 341F and custom barcoded 806R primers, based on primers used in the literature [15]. The PCR used a 2X HotStart Q5 Master Mix (New England Biosciences, Ipswich, MA, USA) and had a final concentration of 0.5 μM of each primer and 0.1 μg/μL BSA with nuclease-free water added to a final reaction volume of 50 μL. The cycling conditions used were as follows: denaturation at 98 °C for 30 s, followed by 30 cycles of 98 °C for 10 s, 64 °C for 20 s, 72 °C for 15 s, and a final elongation step of 72 °C for 2 min. The results of the PCR were run on 1% agarose gels to verify correct amplification. PCR products were then purified with an E.Z.N.A. Cycle Pure Kit (Omega Bio-tek) following manufacturer instructions. Purified PCR products were quantified with a Qubit 2.0 fluorometer and pooled into three libraries with equal mass of the amplicons, before being sequenced on the Illumina MiSeq platform (ICBR, University of Florida, Gainesville, FL, USA) with paired end 2 × 300 sequencing.

### 2.4. Sequence Processing into Amplicon Sequencing Variants

The FASTQ files were returned from the IBCR sequencing already demultiplexed according to the 11-base barcodes on the 806R primers. Demultiplexed FASTQ sequences were pre-processed with Cutadapt to remove primers and adapters, before being analyzed by FastQC for quality [16,17]. In R Studio, DADA2 was used for additional quality trimming, the merging of paired end reads, and the assigning of taxonomy to generate amplicon sequencing variants (ASVs) [18]. Taxonomy assignment was done using the SILVA database (version 138.1) [19,20]. The DADA2 output of ASV and taxonomy tables were, along with sample metadata, imported into the phyloseq R package, which was then used to analyze the composition of the stool samples [21]. At this point, ASVs that were unable to be assigned a kingdom were removed, as well as those with a mean read count of less than 5 across all samples. The MicrobiomeStat R package was used to determine differentially abundant taxa between the sample conditions [22]. *p*-values were adjusted for control of the false discovery rate with multiple testing using the Benjamini and Hochberg method. In addition, the vegan R package “adonis” function was used to test for differences in composition between the MNV and antibiotic conditions with the permutational multivariate analysis of variance (PERMANOVA) test.

### 2.5. Stool Bacterial Extracellular Vesicle Extraction and Removal of Exosomes

Bacterial extracellular vesicles were isolated from stool samples using a modified method that was previously described [13]. Twenty-five mg of stool was homogenized in 500 μL of PBS and centrifuged at 500× *g* for 10 min. The supernatant was transferred to a new tube and the pellet was resuspended in 500 μL PBS. Homogenization, centrifugation, and supernatant transfer were repeated three times to obtain 2 mL of supernatant. The supernatant was centrifuged at 10,000× *g* for 30 min to pellet bacteria, larger particles, and large extracellular vesicles. The remaining supernatant was filtered through a 0.22 μm filter and ultracentrifuged at 110,000× *g* for 2 h at 4 °C in a Beckman Optima XE-90. The supernatant was removed and the pellet containing extracellular vesicles was resuspended in 4 mL of PBS and ultracentrifuged at 110,000× *g* for 70 min at 4 °C. The resuspension and ultracentrifugation were repeated three times to wash away any stool contaminants. The final pellet was resuspended in 500 μL dPBS with a protease inhibitor cocktail. To confirm the filter integrity, 10 μL spots of the isolated extracellular vesicles were plated on LB agar and were checked for bacterial growth.

Following the last ultracentrifuge spin, murine exosomes were removed from the vesicle suspension using the Pan Exosome Isolation Kit (Miltenyi Biotech #130-117-039), which binds exosomes with CD9, CD63, and CD91 tetraspanins, following the manufacturer’s instructions. Microbeads were incubated with the vesicle solution and the mixture loaded onto the kit’s column, which was placed in the kit’s magnetic holder. The exosomes were retained on the magnetic column while the flow-through solution containing bEVs was collected and analyzed by nanoparticle tracking analysis (NTA).

### 2.6. Nanoparticle Tracking Analysis

Bacterial extracellular vesicles were diluted 1:300 in filtered autoclaved water and loaded onto a NanoSight NS300 sizer and counter (Malvern) for nanoparticle tracking analysis (NTA), which determines size and concentration using the Brownian movement of the particles. The analysis was recorded for 60 s per technical replicate, with five technical replicates for each sample and nine biological replicates.

### 2.7. Western Blot

Samples containing either exosomes or stool-derived bacterial extracellular vesicles were lysed with RIPA buffer and then separated by SDS–PAGE on a 4–12% Bis-Tris acrylamide gel with XT-MES running buffer. The separated proteins were then transferred onto a PVDF membrane. The membrane was blocked with 5% nonfat milk followed by incubation with primary and then with secondary antibodies (CD9- 1:1000 and 1:10,000 dilutions, System Bioscience # EXOAB-KIT-1; HSP70- 1:1000 dilution, Invitrogen #MA3-006; Lipid A LPS- 1:1000 dilution, Invitrogen #PA1-73178). The membrane was kept for 5 min in Pierce™ ECL Western blotting substrate (Thermo Fisher). The blot was visualized using iBright (Invitrogen).

### 2.8. Additional Data Analysis and Statistics

One-way ANOVA was used to analyze the NTA results with Tukey’s multiple comparisons test. Statistical significance of the viral titers was measured using two-way ANOVA with Tukey’s multiple comparisons test. Graphs and plots were made using either GraphPad Prism (version 8.0.0 for Windows, GraphPad Software, San Diego, CA, USA) or R Studio (version 1.3.1093 with R version 4.0.2) using ggplot2 [23].

## 3. Results

### 3.1. Study Design

**Figure 1:**
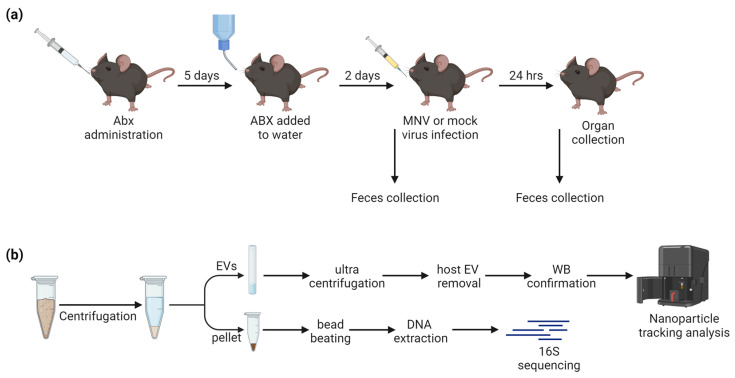
Study design. (**a**) ABX administration followed by MNV infection, with fecal sample collection occurring immediately before and 24 h after MNV or mock inoculum administration. (**b**) Fecal samples were homogenized and centrifuged to separate EVs from the fecal pellet prior to downstream processing.

The mouse study consisted of three experiments, each with three mice per condition for a total of 54 mice (Figure 1a). The mice were treated with either broad-spectrum antibiotics, anaerobe-targeting antibiotics, or PBS as a non-antibiotic control, for 7 days prior to infection with MNV-1 or mock inoculum (Table 1). The stool sample that was collected just prior to MNV-1 infection was the “pre” sample, while, 24 h after MNV-1 infection, the “post” stool samples were collected, for a total of 108 stool samples. After the last stool sample was collected, the mice were euthanized, and the intestines were sectioned for viral titer assays (Figure 1a).

Both pre- and post-infection stool samples were processed for 16S rRNA sequencing. Post-infection fecal samples were additionally used for the isolation of bacterial extracellular vesicles (bEVs). Weighed fecal samples were homogenized in PBS and centrifuged (Figure 1b). The supernatant was used for extracellular vesicle isolation, where host exosomes were removed with anti-tetraspanin-coated beads and confirmed via Western blot. The fecal pellet was retained for 16S sequencing of the microbiome. After sequencing, samples with low sequencing reads overall (<1000) were removed, leaving a total of 101 stool microbiome samples processed. For 16S sequencing, after the quality trimming and filtering steps, there was a median of 291,170 reads (1369–1,107,667 reads). After the removal of low-abundance reads, 677 ASVs were seen in total.

### 3.2. Regionalization of MNV Infection and Increased Bacterial Extracellular Vesicle Production

**Figure 2:**
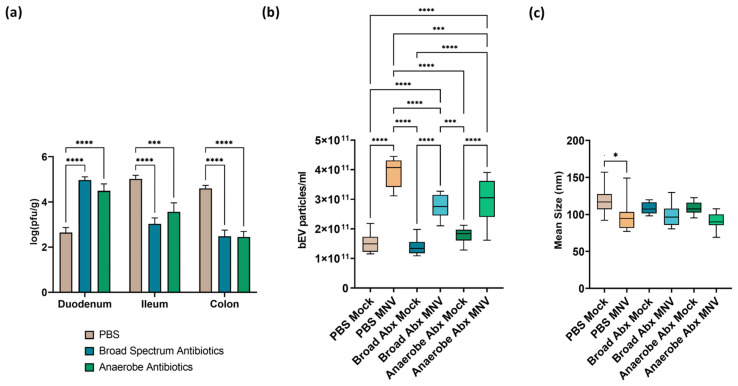
Impact of ABX treatment on MNV infection and bEV production at 24 h post-MNV-infection. (**a**) Plaque assay was used to quantify MNV from the indicated tissues and results normalized to tissue weight. Statistical significance was measured using a two-way ANOVA with Tukey’s multiple comparison testing (*n* = 9 mice per condition). NTA was used to evaluate bacterial extracellular vesicles harvested from 25 mg of stool after removal of murine exosomes by (**b**) quantifying vesicles produced and (**c**) measuring average vesicle size. Statistical significance was determined by one-way ANOVA with Holm–Šídák’s multiple comparisons test (*, *p* < 0.05; ***, *p* < 0.001. ****, *p* < 0.0001). *Broad Abx*, broad-spectrum antibiotic cocktail. *Anaerobe Abx*, anaerobic bacteria-targeting antibiotic cocktail.

The results showed robust MNV titers in the intestine 24 h post-infection (hpi) and demonstrated the regionalization effect of commensal bacteria, which has been previously reported [8], where the presence of commensal bacteria inhibits viral infection in the proximal small intestine while enhancing the MNV infection of the distal regions of the intestinal tract (Figure 2a). Interestingly, the antibiotic depletion of anaerobic bacteria alone did not alter viral replication in any of the intestinal regions examined, compared to viral replication in mice treated with the broad-spectrum antibiotic (Figure 2a). Since both ABX treatments had a similar level of viral replication and both treatments targeted anaerobic bacteria, this result indicates that aerobic bacteria, or at least those not targeted by anaerobe depletion antibiotics, play a role in modulating MNV replication along the intestinal tract, and that the same bacterial populations may be responsible for MNV suppression in the duodenum and MNV enhancement in the ileum and colon.

Feces harvested after MNV infection were used for the extraction of bEVs, which were quantified using nanoparticle tracking analysis (NTA). This analysis showed that the concentration of bEVs was significantly increased in MNV-infected mice compared to the mock-infected mice across the different antibiotic conditions (Figure 2b). However, the mice that received either of the antibiotic cocktails and were also MNV-infected showed significantly decreased amounts of bEVs when compared to the non-antibiotic (PBS) MNV-infected mice. The broad-spectrum antibiotic condition had the lowest amount of bEVs among the MNV-infected and mock-infected conditions. This result indicates that the depleted bacterial populations are involved in the previously reported MNV stimulation of bEV production [8]. Interestingly, there were no differences in bEV amounts between the mock samples regardless of ABX treatment, which is surprising since the microbiome was depleted of culturable gut flora, as determined by plating to blood agar plates [5]. Antibiotics have been previously shown to induce hypervesiculation in bacteria [24,25] and, while not explored in this study, this may explain the similar bEV levels between ABX-treated and untreated mock-infected mice. Alongside vesicle quantification, we also compared the size of bEVs produced among the various treatment and infection conditions. As we previously reported [13], infection with MNV in the presence of the full microflora (PBS condition) revealed a significant decrease in mean bEV size compared to bEVs collected from mock-infected mice (Figure 2c). Interestingly, MNV infection did not significantly alter the average bEV size under either of the antibiotic treatment conditions. Changes in the size of bacterial vesicles can indicate a change in vesicle contents. Since anaerobic bacteria were eliminated in both the antibiotic treatment conditions, this suggests that these bacterial families may be more susceptible to changes in bEV content in response to MNV infection. Future studies examining the proteomic and lipidomic content of vesicles will be necessary to test this hypothesis.

### 3.3. Differences in Diversity Indices Are Explained by Antibiotic Condition

**Figure 3:**
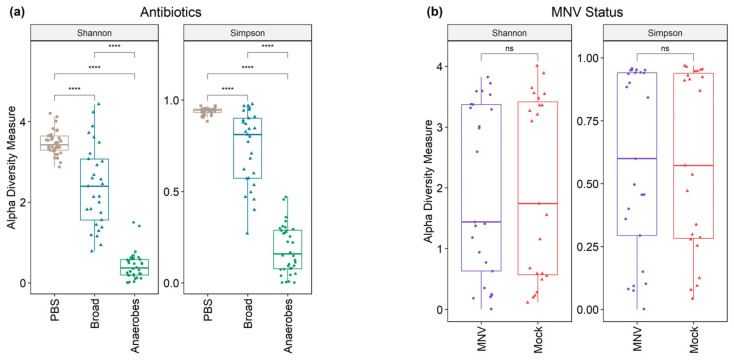
Alpha diversity shows a difference in antibiotic measures but not when grouped by MNV status. (**a**) Alpha diversity (Shannon and Simpson measures) shows significant differences across antibiotic types in mock-infected samples. (**b**) Alpha diversity measures are not significant when looking at the post samples and comparing MNV-infected mice to the mock inoculum mice. Pairwise significance testing was performed with a Wilcox test (****, *p* < 0.0001; *ns* = not significant). *Broad*, broad-spectrum antibiotic cocktail. *Anaerobes*, anaerobic bacteria-targeting antibiotic cocktail.

Following 16S sequencing, the alpha diversity indices of the microbiomes were analyzed, grouped by both antibiotic and MNV conditions (Figure 3). The Shannon and Simpson diversity indices were calculated to measure the alpha diversity. Although the Simpson index places less weight on lower-abundance samples than the Shannon index, the results were similar. Results showed that the alpha diversity differed significantly (Kruskal–Wallis, *p*-values < 2.2 × 10^−16^) among the different antibiotic conditions, with the antibiotic cocktail aimed at targeting anaerobic bacteria having the lowest alpha diversity indices, whereas the non-antibiotic PBS condition had the highest. Pairwise comparison testing between the groups also showed high significance in uninfected (mock) mice (Figure 3a). To investigate whether MNV infection caused a difference in the alpha diversity measure, the samples were subset to only include post-infection samples. After 24 h, MNV infection did not cause significant overall changes to the alpha diversity indices with either measure (Wilcoxon *p*-values = 0.87/0.89 for Shannon and Simpson, respectively; Figure 3b), even in antibiotic-treated mice.

**Figure 4:**
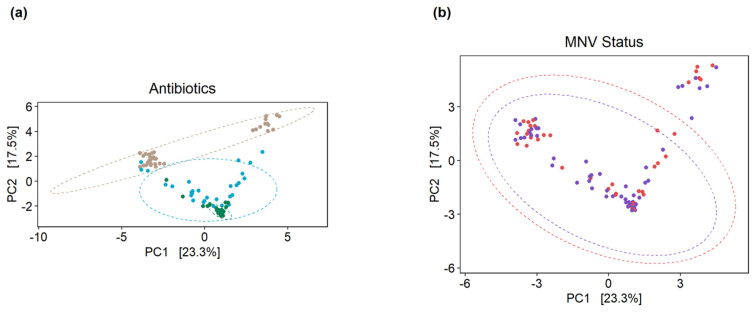
Principal component analysis plots on clr-transformed count data illustrate the beta diversity of the mice microbiomes. (**a**) When grouped by antibiotic condition, clustering of the mock-infected samples can be seen, where the PBS samples (brown circles) are distinct from the two antibiotic conditions, while the anaerobe-depleted condition samples (green circles) are clustered tightly but overlap with the broad condition samples (blue circles). (**b**) Grouping by MNV status does not result in the same separation of samples, where purple circles are samples from MNV infection and red circles are samples from mock infection. *Broad*, broad-spectrum antibiotic cocktail. *Anaerobes*, anaerobic bacteria-targeting antibiotic cocktail.

To measure the beta diversity, principal coordinate analysis plots were used to show the separation according to the Aitchison distance of the center log-ratio transformed counts. The clear separation of the PBS samples could be seen from the antibiotic conditions in stool collected from uninfected (mock) mice, with some overlap between the broad-spectrum and anaerobe-depleted conditions (PERMANOVA, *p*-value = 0.001) (Figure 4a). However, there was no clear separation between the mock and MNV samples, with considerable overlap in each case (PERMANOVA, *p*-value = 0.811) (Figure 4b).

### 3.4. Use of Antibiotic Cocktails during Norovirus Infection Shows Slight Changes in Overall Gut Microbiota Composition

**Figure 5:**
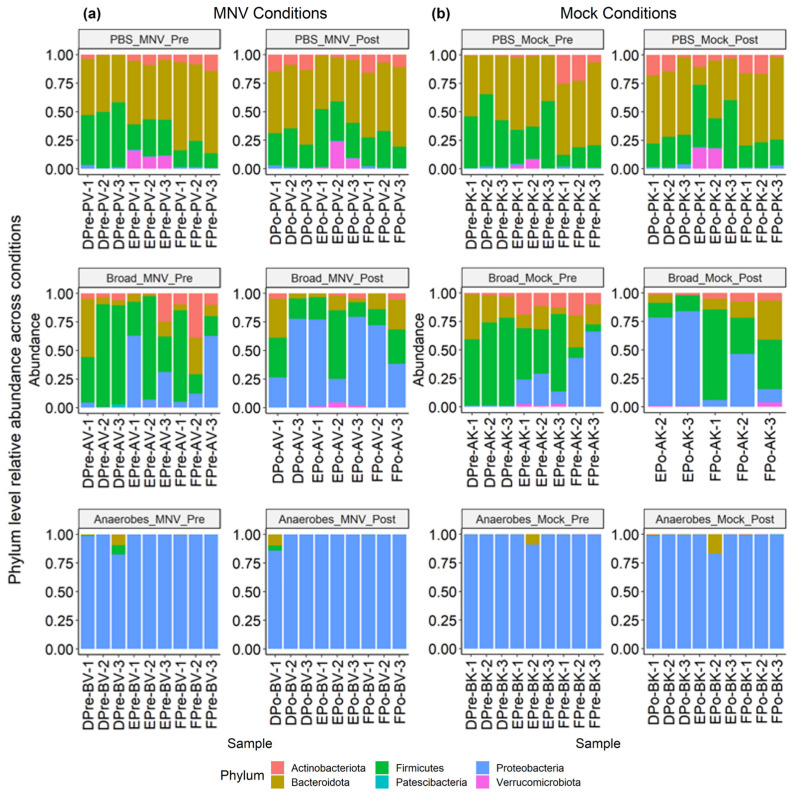
Relative abundance of MNV condition samples and mock condition samples showing both pre- and post-inoculation samples. (**a**) Samples from the MNV-infected mice and (**b**) mock-inoculated mice are grouped by antibiotic condition and pre/post timepoint to show the phylum-level relative abundance per sample. *Broad*, broad-spectrum antibiotic cocktail. *Anaerobes*, anaerobic bacteria-targeting antibiotic cocktail.

This experiment used antibiotic cocktails designed to remove a broad range of bacteria (referred to as the “broad condition”), as well as a cocktail designed to target only anaerobic bacteria (referred to as the “anaerobes condition”). Looking at the overall distribution at a phylum level, we see that the non-antibiotic PBS samples are dominated by Bacteroidota and Firmicutes, for both the MNV and mock samples and in both the pre and post timepoints (Figure 5). For the samples treated with a broad antibiotic cocktail, we see more variability in the composition, with a number of samples showing increased relative abundance of Proteobacteria compared to the non-antibiotic PBS samples. In addition, with the broad-spectrum antibiotic samples, a visual shift from the pre to post conditions can be seen in some samples, primarily consisting of shifts in the ratio of Firmicutes to Proteobacteria. In contrast to both the PBS and broad samples, the samples treated with the anaerobe-targeting cocktail are dominated by Proteobacteria across the board, with minimal shifts in pre vs. post samples (Figure 5). Together, these results indicate that in a depleted but mixed microbiome population, MNV infection can alter the abundance of Proteobacteria populations, but this impact is muted when Proteobacteria are the only population detected. In fact, since these are measures of relative abundance, it may be that, since Proteobacteria were the only detectable populations, changes could not be detected using this measure.

### 3.5. Slight Changes in Microbiome Composition When Comparing Pre vs. Post MNV Samples

**Figure 6:**
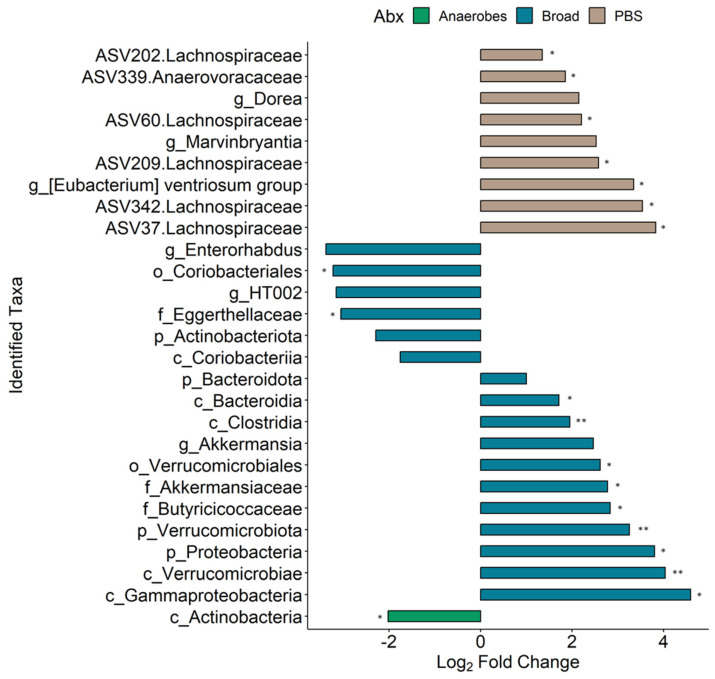
Differential taxa seen between the pre- and post-MNV-infection timepoints. Bars indicate a log_2_ fold change; bar plots are depicted as found using the LinDA model from the MicrobiomeStat R package with a BH-adjusted *p*-value cut-off of 0.1 (*, *p* < 0.05; **, *p* < 0.01) and a log_2_ fold change cut-off of +/−1. The bars are colored based on which antibiotic condition the taxa were found significant in and the taxa names on the y axis include a prefix indicating which taxonomy level is present: p, phylum; c, class; o, order; f, family; g, genus. ASV-level taxa are denoted by the ASV ID followed by the associated family.

To further investigate the changes seen in the MNV condition samples from pre- to post-infection, the R package MicrobiomeStat was used to identify the differential abundance by fitting a linear mixed-effect model, accounting for the experimental batch as a covariate. To have a better understanding of how each antibiotic condition affected any changes seen from the pre to the post conditions for the MNV-infected mice, samples for each antibiotic were run separately through the model and the different taxonomic levels were aggregated and run through the model as well. The combined results can be seen in Figure 6 (values in Supplemental Table S1). As could be expected from the results displayed in Figure 5, the PBS and broad-spectrum antibiotic conditions showed the most changes, with a total of nine differentially abundant taxa for the PBS condition and 17 differentially abundant taxa for the broad condition, compared to only one for the anaerobe-depleted condition. Of those seen to be differentially abundant in the PBS condition, six of the nine taxa were at an ASV level, with five of them belonging to the *Lachnospiraceae* family, while the three genera identified were *Dorea*, *Marvinbryantia*, and the [*Eubacterium*] ventriosum group, suggesting that, without antibiotics, there is a lack of large-scale changes but there are more nuanced shifts. The broad-spectrum condition had a larger breadth of taxonomy levels seen, but, in contrast to the PBS condition, no ASV-level changes were found to be significant. For this condition, there were four phyla (Proteobacteria, Bacteroidota, Actinobacteriota, Verrucomicrobiota), five classes (Gammaproteobacteria, Bacteroidia, Verrucomicrobiae, Clostridia, Coriobacteriia), two orders (Verrucomicrobiales, Coriobacteriales), three families (*Akkermansiaceae*, *Butyricicoccaceae*, *Eggerthellaceae*), and three genera (*Akkermansia*, *HT002*, *Enterorhabdus*). It was also noteworthy that there were no shared taxa across the post to pre comparisons for the three conditions, indicating that changes in overall microbiome composition, such as changes due to antibiotics, may impact the influence of MNV infection on bacterial abundance.

### 3.6. Comparisons of Post-MNV-Infection Samples Show Antibiotic-Driven Differences in the Microbiome

**Figure 7:**
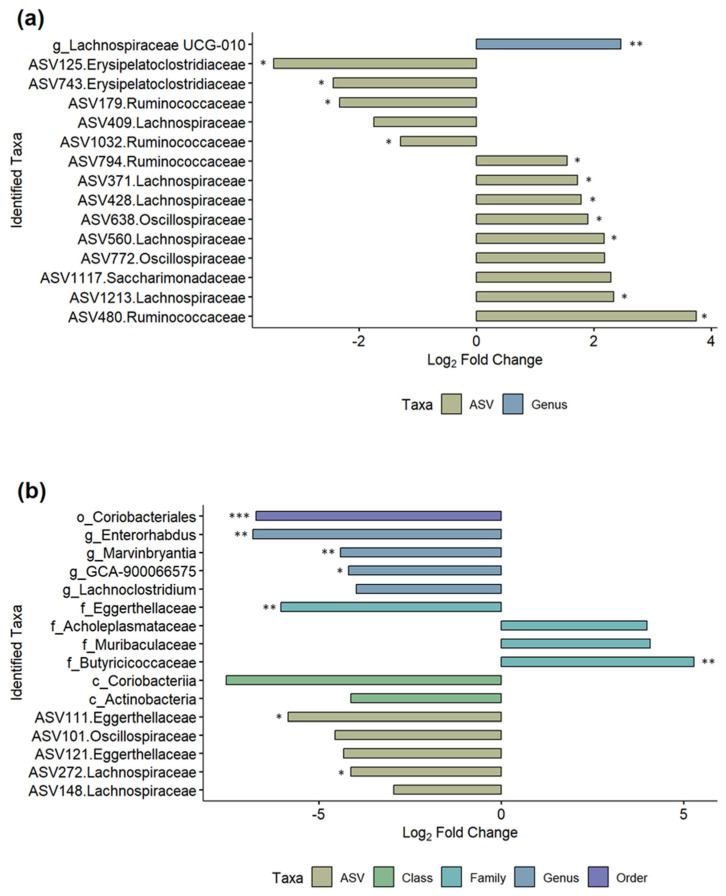
Differential taxa seen between the MNV and mock conditions for the post timepoints. Log_2_ fold change bar plots are shown for (**a**) the PBS condition between MNV vs. mock and (**b**) the broad antibiotic condition for MNV vs. mock. Both graphs show the differentially abundant taxa found using the LinDA model from the MicrobiomeStat R package with a BH-adjusted *p*-value cut-off of 0.1 (*, *p* < 0.05; **, *p* < 0.01; ***, *p* < 0.001) and a log_2_ fold change cut-off of +/−1. The bars are colored based on the taxonomy levels and the taxa names also indicate the taxonomy levels: c, class; o, order; f, family; g, genus. ASV-level taxa are denoted by the ASV ID followed by the associated family.

We further investigated which microbiome changes were seen between the MNV- and mock-infected mice after MNV or mock infection in order to gain a better understanding of which bacteria may be responsible for driving the increased bEV production seen in Figure 2b. Each of the three antibiotic conditions was modeled separately using the MicrobiomeStat R package (Figure 7, Supplemental Table S2). Comparing PBS MNV to PBS mock showed 15 differentially abundant taxa (Figure 7a), with 14 of 15 being at the ASV level, similar to the results for the post vs. pre PBS MNV condition seen in Figure 6. Of the 14 ASV-level results, five of the ASVs belong to the *Lachnospiraceae* family, and four to the *Ruminococcacaea* family, with the remaining five split between the *Erysipelatoclostridiaceae*, *Oscillospiraceae*, and *Saccharimonadaceae* families. The differentially abundant genus was *Lachnospiraceae* UCG-010. For the broad-spectrum MNV vs. broad-spectrum mock at the post samples, 16 differentially abundant taxa were seen (Figure 7b). Similar to the broad-spectrum MNV post vs. pre samples, the taxonomy levels are more varied for the broad condition compared to the PBS condition, with one order (Coriobacteriales), two classes (Actinobacteria, Coriobacteriia), four families (*Muribaculaceae*, *Acholeplasmataceae*, *Eggerthellaceae*, *Butyricicoccaceae*), four genera (*Lachnoclostridium*, *Marvinbryantia*, *GCA-900066575*, *Enterorhabdus*), and five ASV-level taxa identified as differential. At the ASV level, the *Lachnospiraceae*, *Eggerthellaceae*, and *Oscillospiraceae* families are represented. The anaerobe MNV vs. anaerobe mock resulted in a single phylum-level result for Firmicutes; however, this appeared to be mainly driven by a single sample when looking at the bar graphs (Figure 5) and it is not shown in Figure 7. These results highlight the ability of antibiotic treatment to illuminate the impacts of viral infection on bacterial taxa abundance.

**Figure 8:**
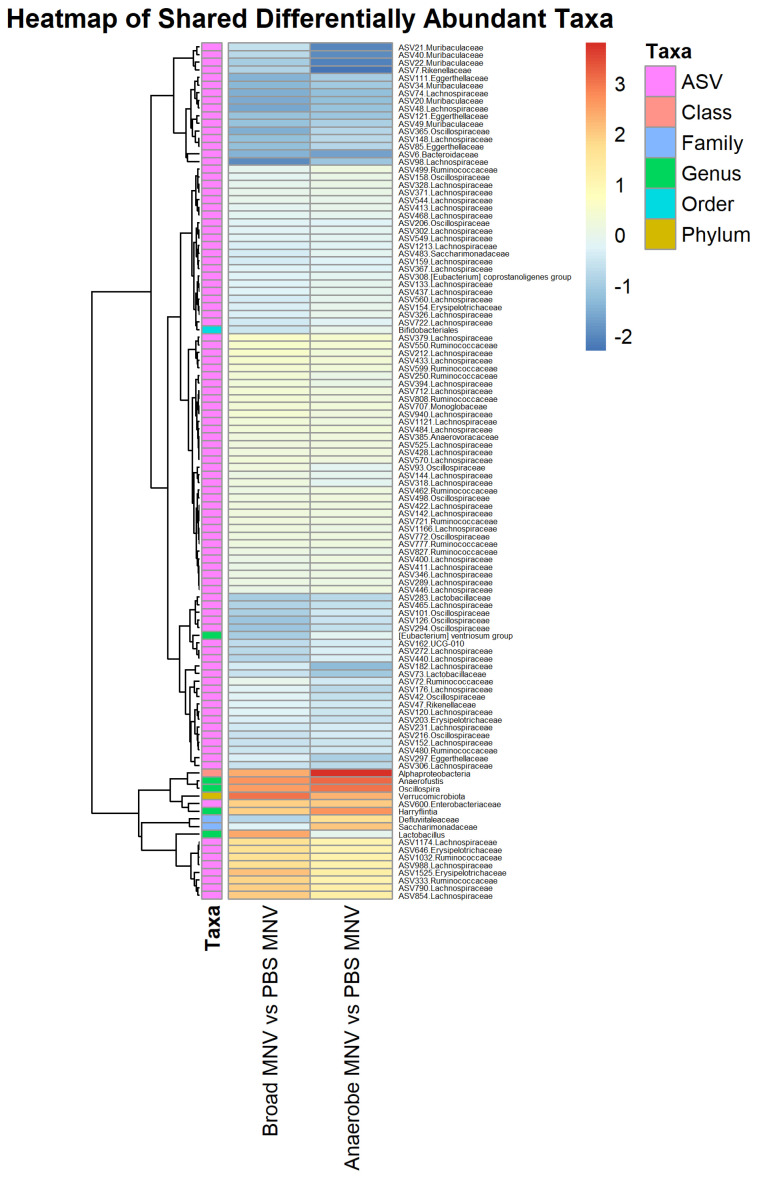
Shared trends are seen in the differentially abundant taxa of the antibiotic MNV samples vs. the PBS MNV samples. Heatmap showing the scaled log_2_ fold changes for the 112 identified taxa across multiple taxonomy levels that were shared between the two comparisons of antibiotic MNV mice vs. PBS MNV mice. The majority of the shared taxa also share whether they are increased or decreased in abundance compared to the PBS MNV samples.

Another point of interest was to determine which differentially abundant taxa were present in the antibiotic MNV samples compared to the PBS MNV samples, and, based on the visual overview in Figure 5, it was expected that taxa would be found to be differential when comparing these groups. For the broad-spectrum MNV vs. PBS MNV post samples, a total of 281 taxa were found to be differentially abundant. These were distributed across four phyla, five classes, seven orders, 12 families, 23 genera, and 230 at the ASV level (Supplemental Table S3). Nearly half of the differentially abundant ASVs (111) belonged to the *Lachnospiraceae* family. When comparing the anaerobe MNV vs. the PBS MNV at the post timepoint, 352 taxa were found to be differentially abundant. This included four phyla, four classes, 11 orders, 16 families, 35 genera, and 282 at the ASV level (Supplemental Table S4). Similar to what was seen in the broad-spectrum MNV vs. PBS MNV comparison, nearly half (135) of the differential ASVs belonged to the *Lachnospiraceae* family. There were 252 identified taxa that were shared between the two comparisons of antibiotic MNV mice vs. PBS MNV mice; however, when the taxa shared between the broad-spectrum mock and anaerobe mock vs. the PBS mock were removed, there were 112 taxa whose differential abundance could not be explained by the antibiotics alone (Figure 8). Since these changes are not accounted for by the antibiotics, they are likely the result of infection. These taxa include one each at the phylum, class, and order levels, along with 2 families, 5 genera, and 102 ASVs. Of these 112 shared taxa, 14 share increased abundance and 95 share decreased abundance compared to PBS MNV (Supplemental Table S5). For each treatment comparison, Table 2 displays the top five taxa that are increased or decreased in abundance and the log fold changes compared to the PBS treatment.

## 4. Discussion

Both commensal bacteria and the extracellular vesicles that they produce have been shown to influence norovirus infection [5,8,12]. To better define the bacterial populations involved in modulating MNV infection, antibiotic depletions were performed to broadly or selectively reduce the bacterial populations in the intestinal tract. Bacterial extracellular vesicles were also isolated and quantified to determine if correlations between changes in bacterial population, MNV infection, and bEV production existed.

Previous studies have compared the microbiome between MNV-infected and mock-infected murine populations and found differing results. Specifically, Hickman et al. [6] found significant changes at the phylum level when comparing stool samples collected from MNV-1-infected mice at 0 and 5 days post-infection. Conversely, Nelson et al. [7] found no significant changes in microbiome composition at the peak of viral replication (24 hpi). In our study, we examined the microbiome pre- and post-infection to determine which bacterial families dominated the murine microbiomes both before and after MNV-1 infection, with and without the presence of antibiotic cocktails, and delved deeply into changes in taxa abundance in the presence and absence of treatment. In contrast to the previously published works, our results showed specific changes in microbiome composition after MNV infection in mice that did not receive ABX treatment (e.g., PBS mice), particularly at more specific taxonomic levels. For example, when comparing the post-MNV-infection samples with the pre-infection PBS samples, the results showed a few differentially abundant ASVs and genera. These samples did not reveal any higher-level changes at the phylum level, which was consistent with previously published reports [7]. However, in mice treated with broad-spectrum antibiotics, MNV-1 infection altered the microbiome composition at all taxonomic levels. The anaerobe-depleted condition showed the fewest changes after infection, with primarily one phylum contributing to the bacterial abundance noted. The extreme abundance of Proteobacteria did not allow for a clear evaluation of the relative abundance of minor bacterial populations. It is possible that populations in minor abundance could impact viral replication and a repeat of these experiments with the future inclusion of additional antibiotic cocktails is needed. This study was limited by the amount of stool produced by the mice at each sampling time. Future studies could also include larger numbers of mice and the pooling of stool samples to allow for deeper sequencing depths and the possible identification of minor populations. These studies would aid in the identification of these low-abundance populations to ascertain the role, if any, that they play in bEV production or MNV infection.

Interestingly, the effect of MNV infection on the intestinal microbiome composition acts on more taxonomic groups when the microbiome is disturbed by ABX treatment prior to infection but maintains the phylum diversity. Clearly, MNV infection does not significantly impact Proteobacteria as no changes were observed in ASV abundance in anaerobe-depleted bacteria. However, viral infection when all types of bacterial populations are broadly reduced does shift the relative abundance of microbial taxa. This may indicate that ABX-resistant bacteria are more susceptible to stressors induced by MNV infection. It is also possible that these changes occur in PBS-treated mice but are masked by the abundance of the intact microbiota.

We further investigated comparisons between the MNV and mock samples at the 24 hpi timepoint. Our results showed that an increase in bEV amount was present in the MNV samples compared to their respective mock controls for all three treatment conditions. Based on this, and despite no major shifts in bacterial composition, we compared the 16S data for each MNV and mock pairing to determine if shared changes in the bacterial composition could explain this increase in bEVs. This analysis revealed different trends across the three conditions that mirrored the composition of the post vs. pre MNV changes. PBS MNV vs. PBS mock had most of the changes at the ASV level, while broad-spectrum MNV vs. broad-spectrum mock showed changes across taxonomy levels. Meanwhile, anaerobe MNV vs. anaerobe mock only had one hit, which, when looking at the relative abundance bar graphs, appeared to be driven primarily by a single sample, indicating that MNV infection did not alter the microbiome composition in this condition. While there was no exact match in the differential taxa shared amongst these comparisons, at the ASV level, both the PBS MNV vs. PBS mock and the broad-spectrum MNV vs. broad-spectrum mock had ASVs that belonged to the *Lachnospiraceae* and *Oscillospiraceae* families. This suggests that the increases in bEV production may not result from dramatic changes in the overall microbiome composition, but rather smaller shifts in the abundances of existing bacteria following MNV infection, as evidenced by the PBS MNV vs. PBS mock differential taxa consisting mainly of ASV-level results. A complicating factor is the fact that some bacteria increase bEV production under antibiotic stress [26,27,28]. This leaves open the possibility that the drivers of the increased bEVs seen in the antibiotic-treated mice may be the result of antibiotic-driven stress, in addition to the stress shown to be induced by commensal bacteria upon interactions with MNV [13]. Therefore, the drivers of increased bEV production observed during MNV infection in antibiotic-treated mice may be different from those driving the increased bEV production in the PBS mice.

It is also worth noting the significance of changes in the abundance of the *Lachnospiraceae* because of viral infection. These bacteria are among those abundantly found in the mammalian intestinal tract throughout life [29,30] and are known to play a significant role in carbohydrate metabolism [31,32]. Importantly, these bacteria are among the primary producers of butyrate and other short-chain fatty acids (SFCAs) [32]. In particular, SFCA activity can alter the microbial environment and directly interact with the host immune response [33]. The ability of these compounds to modulate the host immune response could directly impact MNV infection. These taxa were found in decreased abundance in both ABX-treated groups after MNV infection compared to the PBS treatment condition. This could possibly then result in the decreased presence of SFCAs in ABX-treated, MNV-infected mice. The role of SFCAs in norovirus infection has never been explored; however, other bacterial-produced metabolites, such as bile acids, have been shown to influence norovirus infection [8]. The bile acid priming of Type III Interferon leads to the suppression of MNV replication in the proximal areas of the small intestine. While this precise mechanism may not be responsible for the changes in MNV infection levels in other areas of the intestinal tract, it establishes the possibility that SFCAs could play a role in modulating viral infection in more distal regions of the intestinal tract.

Finally, we investigated the differentially abundant taxa between the broad-spectrum MNV and anaerobe MNV vs. the PBS MNV samples. We saw that the bEV production in the antibiotic-treated and MNV-infected mice was higher than the mock samples, but it was lower for both the broad-spectrum and anaerobe-depleted conditions compared to the PBS MNV bEV amounts. We hypothesized that there would be shared bacteria that were different between the antibiotic MNV conditions compared to the PBS MNV condition. This held true in that 112 taxa were identified as shared between the two comparisons, with the majority of these identified at the ASV level. We also saw that of these shared taxa, most of them shared the same differentially abundant direction, with the majority showing a decrease in abundance compared to the PBS MNV samples. Some of these bacteria, especially those that were decreased compared to the PBS MNV condition, could be related to the decreased bEV production compared to PBS MNV bEV production. Moreover, we also used Spearman’s correlation test with Bonferroni–Hochberg correction to investigate whether the relative abundance of individual ASVs correlated to the bEV particles/mL, to further determine the potential drivers of the increased bEV concentration. However, no significant adjusted *p*-values were seen with this correlation test. Further experimentation using different antibiotic treatment regimens would help to elucidate which bacteria are driving the bEV production changes upon MNV infection.

In summary, these data have shown that MNV-1 infection in vivo results in increased bEV production compared to mock inoculum, and that the PBS MNV condition results in larger amounts of bEV production compared to both the broad-spectrum antibiotic MNV condition and the anaerobic-targeting antibiotic condition. When analyzing the 16S sequencing results, nuanced changes were seen in the PBS samples, both in comparing post-infection to pre-infection and comparing 24 hpi MNV samples to mock samples. The broad-spectrum condition showed a more robust shift in the microbiome, with changes seen at multiple taxonomy levels, while the anaerobic-targeting condition displayed the least amount of change due to MNV infection. Ultimately, the results presented herein provide a solid foundation for determining the specific group(s) of bacteria responsible for changes in bEV production in response to infection.

## Figures and Tables

**Table 1:** Antibiotics used in the mouse experiments.

Antibiotic Cocktail	Targeted Bacterial Population (s)	Antibiotic	Administration Amount	Drinking Water Amount
Broad Abx	Gram-negative, Gram-positive, aerobes, anaerobes	Ampicillin	10 mg	1 g/L
		Neomycin	10 mg	1 g/L
		Metronidazole	10 mg	1 g/L
		Vancomycin	10 mg	500 mg/L
Anaerobic Abx	Anaerobes	Metronidazole	10 mg	1 g/L
		Clindamycin	2 mg	1 g/L

**Table 2:** Log2 fold change values for the top five taxa that were increased and decreased in abundance for broad vs. PBS and anaerobe vs. PBS after MNV infection in all conditions.

Taxa ID	Broad vs. PBS	Anaerobe vs. PBS
**Increased Abundance**		
Verrucomicrobiota	4.195023523	5.278166274
Anaerofustis	3.402296713	8.084034485
Oscillospira	3.121227438	7.798614837
Lactobacillus	2.837273213	−2.939306235
Alphaproteobacteria	2.796732207	10.3676823
Harryflintia	1.818983454	6.593577373
**Decreased Abundance**		
ASV21.Muribaculaceae	−4.065631202	−9.578255593
ASV40.Muribaculaceae	−4.353977958	−9.269787528
ASV7.Rikenellaceae	−4.565836714	−10.45958671
ASV22.Muribaculaceae	−4.94867859	−9.72542094
ASV6.Bacteroidaceae	−5.794439552	−8.174547192
ASV74.Lachnospiraceae		−6.858943809
ASV365.Oscillospiraceae	−6.021839134	−5.256030931
ASV20.Muribaculaceae	−6.202646373	−6.744189982
ASV48.Lachnospiraceae	−6.271731816	−6.547431892
ASV98.Lachnospiraceae	−7.061084208	−6.343099048

## Data Availability

Data presented in this study are openly available in Mendeley as Jones, Melissa (2023), “16S sequencing of PBS and ABX treated mice before and after MNV infection”, Mendeley Data, V1, doi: https://doi.org/10.17632/7bj32j5dhj.1.

## Supplementary Materials

The following supporting information can be downloaded at: https://www.mdpi.com/article/10.3390/v15122443/s1, Table S1: Fold changes and *p*-values for the post vs. pre MNV samples; Table S2: Fold changes and *p*-values for the comparisons of MNV vs. Mock in the post samples; Table S3: Fold changes and *p*-values for the comparison of Broad MNV vs. PBS MNV in the post samples; Table S4: Fold changes and *p*-values for the comparison of Anaerobe MNV vs. PBS MNV in the post samples; Table S5: Fold changes used for creation of Figure 8 heatmap showing shared differentially abundant taxa.
